# Electrocatalytic Degradation of Rhodamine B Using Li-Doped ZnO Nanoparticles: Novel Approach

**DOI:** 10.3390/ma16031177

**Published:** 2023-01-30

**Authors:** Vanga Ganesh, Bandapelli Ravi Kumar, Thekrayat. H. AlAbdulaal, Ibrahim. S. Yahia, Mohamed Sh. Abdel-wahab, Ramesh Ade, Mai S. A. Hussien, Mohamed Keshway

**Affiliations:** 1 Laboratory of Nano-Smart Materials for Science and Technology (LNSMST), Department of Physics, Faculty of Science, King Khalid University, P.O. Box 9004, Abha 62529, Saudi Arabia; 2 Department of Physics, Indian Institute of Science, Bangalore 560012, India; 3 Nanoscience Laboratory for Environmental and Bio-Medical Applications (NLEBA), Metallurgical Lab.1., Department of Physics, Faculty of Education, Ain Shams University, Roxy, Cairo 11757, Egypt; 4 Research Center for Advanced Materials Science (RCAMS), King Khalid University, P.O. Box 9004, Abha 61413, Saudi Arabia; 5 Materials Science and Nanotechnology Department, Faculty of Postgraduate Studies for Advanced Sciences, Beni–Suef University, Beni–Suef 62511, Egypt; 6 Department of Physics, Koneru Lakshmaiah Education Foundation, R V S Nagar, Aziz Nagar (P.O.), Moinabad Road, Hyderabad 500075, India; 7 Department of Chemistry, Faculty of Education, Ain Shams University, Roxy, Cairo 11757, Egypt; 8 Egyptian Petroleum Research Institute, 1 Ahmed El-Zomor Street, Nasr City 11727, Egypt

**Keywords:** Li-doped zinc oxide, nanoparticles, optical/electrical properties, electrocatalysis degradation

## Abstract

In this paper, we discuss the preparation of Li-doped ZnO nanostructures through combustion and report on their structural, morphological, optical, and electrocatalysis properties. X-ray diffraction analyses show that the samples have a structure crystallized into the usual hexagonal wurtzite ZnO structure according to the *P63mc* space group. The scanning electron microscope images conceal all samples’ nanosphere bundles and aggregates. The reflectance spectra analysis showed that the direct bandgap values varied from 3.273 eV (for pure ZnO, i.e., ZnL1) to 3.256 eV (for high Li-doped ZnO). The measured capacitance concerning frequency has estimated the variation of dielectric constant, dielectric loss, and AC conductivity against AC electric field frequency. The dielectric constant variations and AC conductivity are analyzed and discussed by well-known models such as Koop’s phenomenological theory and Jonscher’s law. The Raman spectra have been recorded and examined for the prepared samples. Rhodamine B was electro-catalytically degraded in all prepared samples, with the fastest time for ZnL5 being 3 min.

## 1. Introduction

It is common knowledge that all humans rely heavily on natural resources such as soil, water, and air. Industrial processes in the fabric, leatherette, chemical laboratory, and paper sectors have significantly contributed to water contamination in recent years by discharging untreated wastewater into bodies of water. Many lives have been lost and many more harmed due to the use/drinking of dangerous, untreated water. Wastewater treatment and recycling are currently receiving much attention [1,2,3]. As a result, research on cost-effective and environmentally friendly water purifying techniques must be prioritized. Electrocatalysis and photocatalysis are inexpensive and straightforward water purification processes [4].

Due to their high exciton binding energy (60 meV), broad optical band gap (3.37 eV), and other attractive optical and electrical properties, ZnO nanoparticles are a promising II–VI semiconductor material [5]. ZnO is ideal for electronics [6], solar cells [7], UV laser diodes [8], light-emitting diode sensors [9], piezoelectric transducers [10], and optoelectronic devices [11]. Sol-gel [12], ultrasonic [13], chemical vapor deposition [14], microemulsion [15], solvothermal [16], spray pyrolysis [17], electrodeposition [18], sonochemical [19], microwave-assisted [20], hydrothermal [21], and the green synthesis method [22,23] are some of the methods used to make ZnO nanoparticles.

Scientists studying the electrocatalytic performance of different ZnO crystal surfaces have found that the photostability and electrocatalytic activity of the material are profoundly influenced by the surface atomic configurations of the material. Photolysis occurs very quickly when the ZnO surface is polar and has high surface energy. Adding dopants such as transition metals to the ZnO lattice to improve electrocatalytic characteristics has recently been reported [24,25,26]. For example, lithium (Li) has been used for decades to treat psychiatric disorders and bipolar disorder without threat to humans under suitable concentrations [27,28]. Li^+1^ has an ionic radius of 76 Ȧ, which matches with Zn^+2^ (74 Ȧ) and makes viable the incorporation of Li^+1^ into ZnO crystal structures [29,30]. Furthermore, incorporating Li^+1^ in the ZnO lattice has been reported to affect ZnO’s optical properties and reactive oxygen species generation capability [31]. Since these properties are crucial for wastewater treatment, ZnO is a suitable candidate.

Combustion synthesis of advanced materials is an energy-efficient synthesis process. It occurs through two steps. The first is self-propagation. An external source locally preheats a reactive medium to the ignition temperature, causing a reaction in this layer. The “hot” reacting layer preheats and ignites the following “cold” layer, which self-propagates the combustion front and forms the desired solid product [32]. It gives a choice of molecular precursor and can influence a material structure even after the solution or gel has been dried and heated. For this, a strongly exothermic reaction between an oxidant such as nitrate and fuel such as citrate or glycine produces large volumes of gas that result in an open foam-like structure [33]. Khorsand et al. [34] successfully prepared plate-shaped zinc oxide nanoparticles (ZnO-NPs) using the combustion method. Nooria et al. [35] used a gel combustion method to prepare ZnO nanopowder with different fuels of glycine, urea, and citric acid with various ratios of fuel to salt and different primary pH values, followed by calcination at temperatures of 400–600 °C. They concluded that using citric acid at a calcination temperature of 500 °C enhanced ZnO’s structure and morphological structure.

This paper shows how to produce pure and Li-doped ZnO nanoparticles using a low cost-effective combustion synthesis approach. First, different techniques were used to characterize the prepared samples to show their structural, morphological, and optical properties. Then, all prepared samples were electro-catalytically degraded under optimal conditions with rhodamine B, a pollutant example for wastewater. RhB dye’s cancer-causing properties can irritate the eyes and skin while posing risks to the respiratory, reproductive, and nervous systems. Moreover, the necessity of treating the RhB effluent increases since rhodamine B is toxic even at low concentrations [36].

## 2. Experimental Conditions

### 2.1. Preparation of Li-Doped ZnO Nanoparticles

In the present work, pure and lithium-doped zinc oxide nanoparticles were prepared by a low-cost combustion method. First, as starting material, 5 g of zinc acetate Zn(CH_3_CO_2_)_2_.2H_2_O and 5 g of citric acid H.O.C. (CH_2_CO_2_H)_2_ were weighed in the crucible and mixed well with a stirrer. Then, different concentrations of Lithium nitrate (LiNO_3_) (without glycine of 0 wt.%, with glycine of 0 wt.%, 0.001 wt.%, 0.01 wt.%, 0.1 wt.%, and 0.5 wt.%) were added to the above-mixed materials separately. Next, 30 mL of distilled water was added to the well-mixed powders and continuously stirred for 2 h on a hot plate at 170 °C. Finally, these crucibles were transferred to the well-programmed furnace and heated for 2 h at 550 °C. After heating the furnace, the final product was ground using a mortar and pestle. The sample details and their codes are listed in Table 1.

### 2.2. Devices and Instruments

Different experimental techniques characterized pure and lithium-doped zinc oxide nanoparticles in the present work. First, the structural studies of prepared samples were described by using an X-ray diffractometer (XRD) using Shimadzu LabX-XRD-6000 (Kyoto, Japan) by exploiting filtered radiation of *CuK_α_* (λ = 1.5406 Ǻ) at room temperature at diffraction angles (2θ) ranging from 5° and 70° with a step size of 0.02°. Then, the XRD results were analyzed with the programmed software (XRD-6000 – LabWrench) (pdf-2 library, available online: https://photos.labwrench.com/equipmentManuals/7650-2830.pdf, accessed on 1 April 2022) in XRD Shimadzu.

The scanning electron microscopy (SEM) technique (Jeol. JSM-6360 type (Akishima, Japan) with a 20 kV operating voltage) was used to study the morphology of prepared nanostructured materials. To obtain the optical bandgap of the prepared samples, the optical diffused reflectance spectra were recorded using a 3600 UV/Vis/NIR spectrophotometer (Shimadzu, Japan) in the wavelength range from 300 to 800 nm with a step size of 5 nm.

FT-Raman spectrometer (Thermo Fisher Scientific, Waltham, MA, USA) was used to detect the presence of secondary phase modes in the as-prepared nanostructured materials.

The dielectric properties of prepared samples were measured using a computerized digital Keithley 4200-SCS (Cleveland, OH, USA) with a broad range of frequencies between 0.1 MHz and 10 MHz.

### 2.3. Electrochemical Degradation Experiment

The electrochemical oxidation was carried out in a two-electrode electrochemical cell with two graphene electrodes, and 0.01 g of pure and Li-doped ZnO nanoparticles was added to 200 mL of aqueous solution Rh. B (50 ppm) dye, followed by 10 mL of NaCl 1 M. Two graphite rods form the working electrode. Germany supplied each graphite rod with a length of 25 cm and a diameter of 1 cm. The electrodes are 5 cm apart and immersed in the dye solution, with a D.C. voltage of 10 V from a 6 A power supply (Phywe business, Göttingen, Germany). In addition, a 3600 UV/Vis/NIR spectrophotometer (Shimadzu, Japan) in the wavelength range from 300 to 800 nm with a step size of 5 nm was used to follow up the photoelectrodegradation of the RhB solution by measuring the withdrawn solution after an interval time of irradiation until complete degradation.

## 3. Results and Discussion

### 3.1. XRD Studies of Li-Doped ZnO Nanoparticles

**Figure 1:**
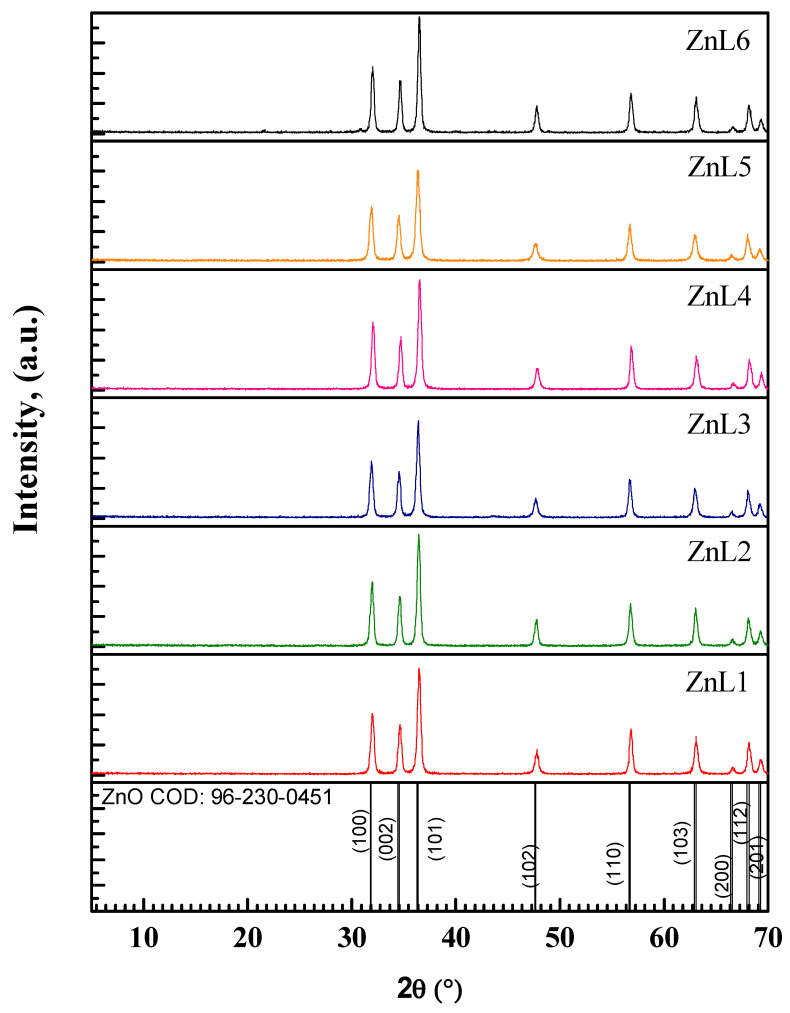
X-ray diffraction patterns of the pure and Li-doped ZnO nanostructure.

To obtain the crystalline nature of the prepared pure and Li-doped ZnO nanoparticles, X-ray diffraction (XRD) measurements were performed at room temperature. The corresponding patterns are displayed in Figure 1. This figure indicates that all the prepared samples crystallized into a standard hexagonal wurtzite ZnO structure [JCPDS file No: 01-089-1397] with a *P63mc* space group [37]. All the samples exhibit polycrystalline nature, and the observed Bragg’s peaks in the 2*θ* range between 5°–70° could be indexed with (100), (002), (101), (102), (110), (103), (200), (112) and (201) planes (from left to right), according to the above-mentioned hexagonal wurtzite ZnO structure. The absence of any other Bragg’s peaks reveals that the doping of Li in the host ZnO lattice leaves its crystal structure unaltered. The XRD pattern for the present samples almost matches our previously reported XRD pattern of Ti-doped ZnO thin films [38]. The structural parameters, such as lattice parameters (*a = b*, *c*), unit cell volume (*V*), average crystallite size, strain (*ε*), and dislocation density (*δ*), are estimated using the relations described below. The values of *a* (*=b*) and *c* for <100> and <002> planes, respectively, are deduced by [39]:

(1) $$a=\frac{\lambda }{\sqrt{3}sin\theta }$$

(2) $$\mathrm{and}\;c=\frac{\lambda }{sin\theta }$$

The value of *V* is calculated using the relation [39]:

(3) $$V=\frac{\sqrt{3}}{2}a^{2}c$$

Here, *d* and (*h k l*) indicate the d-spacing and Miller indices. Scherrer’s formula calculates the *D* value for (101) planes [39]:

(4) $$D=\frac{K\;\lambda }{\beta_{2\theta }\;cos\theta }$$

*K* denotes the shape factor, 0.94, and $\beta_{2\theta }$ is FWHM. The *ε* value is calculated by [40]:

(5) $$\varepsilon =\frac{\beta_{2\theta }\;cot\theta }{4}$$

The dislocation density *δ* is calculated by the following equation [41]:

(6) $$\delta =\frac{1}{D^{2}}$$

Table 2 summarizes the structural parameters calculated by Equations (1)–(6). As is evident from this table, Li-doping in ZnO resulted in an expansion of the lattice for the samples up to ZnL3. Still, beyond this Li-concentration, the doping is accompanied by a lattice contraction first, then an increase, and finally a contraction again. These may be ascribed to the fact that the Li-ions may be settled into interstitial positions whenever there is a lattice expansion.

In contrast, Li-ions may prefer to substitute Zn-sites because Zn and Li ions have almost the same ionic radii of 0.60 nm [42]. As a result, D, *ϵ*, and *δ* values do not change significantly but fluctuate around some value for all the samples. However, a relatively lower *D* (higher *ϵ* and *δ*) value is observed for ZnL5.

### 3.2. Surface Morphology of Li-Doped ZnO Nanoparticles

**Figure 2:**
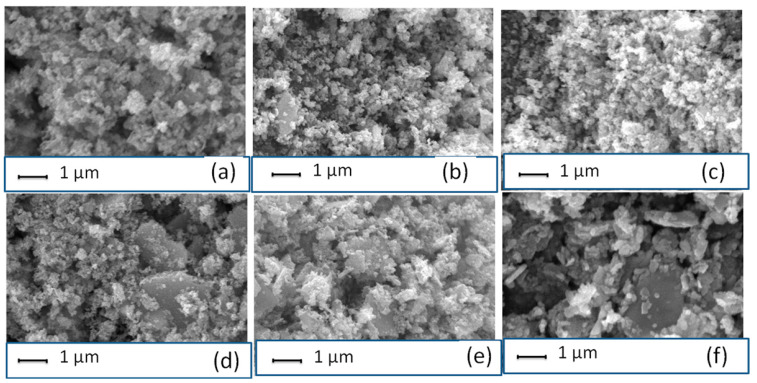
SEM micrographs of (**a**) ZnL1, (**b**) ZnL2, (**c**) ZnL3, (**d**) ZnL4, (**e**) ZnL5, and (**f**) ZnL6 nanostructures.

SEM. micrographs have been recorded to capture the surface morphologies of the prepared samples. As illustrated in Figure 2a–f, all the samples have nanosphere bundles and aggregates. Furthermore, ZnL5 and ZnL6 have different microstructures and a few nano rod-like structures coexisting with the nanosphere bundles. Nevertheless, the average crystallite size and morphologies of all the samples qualitatively agree, and this agreement is revealed by comparing the micrographs with the XRD results.

### 3.3. Dielectric and A.C. Electrical Properties of Li-Doped ZnO Nanoparticles

Dielectric response of solids can be obtained by utilizing an expression relating the complex relative dielectric constant with real (*ε*_1_) and imaginary parts (*ε*_2_), given by [43]:

(7) $$\epsilon^{*}=\varepsilon_{1}+i\varepsilon_{2}$$

The *ε*_1_ measures the energy stored in the material from the applied electric field, whereas the *ε*_2_ describes the dielectric loss or dissipation energy. To extract the dielectric constant, the following equation is used [44]:

(8) $$\varepsilon_{1}=\frac{C\times t}{A\varepsilon_{0}}$$

**Figure 3:**
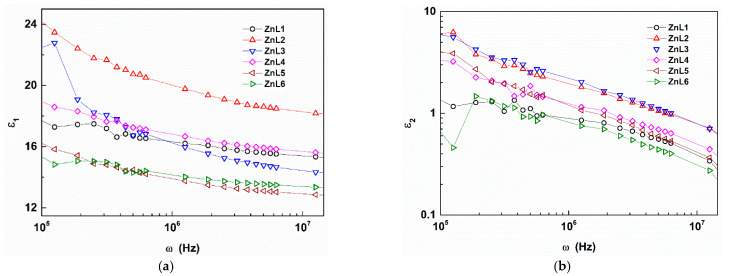
(**a**) The Dielectric constant as a function of the applied frequency and (**b**) double logarithmic variation of dielectric loss with frequency.

Here, *C* is capacitance, *t* is the thickness, A is the pellet sample’s cross-sectional area, and *ε*_0_ is the vacuum permittivity. Thus, the calculated dielectric constant from Equation (8) is plotted against frequency for all the samples at room temperature in Figure 3a. This figure highlights that the dielectric constant decreases within the frequency range with increased frequency. This decrease validates Koop’s phenomenological theory [45] and the Maxwell–Wagner polarization model, which describes the dielectric nature of conducting grains layered with resistive grain boundaries. Upon applying an external electric field, the charge carriers migrate within the grains and pile up at the grain boundaries, resulting in large interfacial/space charge polarization within the dielectric medium, thereby creating a high dielectric constant at low frequencies [46]. The reduction of the dielectric constant after attaining the peak with an increase in frequency may be due to the hopping of the charge carriers that lags behind the alternating electric field [47]. Next, the dielectric loss tangent or loss factor can be calculated by the relation [48] *tanδ = ε*_2_*/ε*_1_, where *δ* is the phase difference between the electric field and the resultant polarization of the dielectric material. Like the dielectric constant, the dielectric loss tangent also reduces with the frequency, which appears to follow a power law, an inversely proportional relation between *tanδ* and *ω* [49], as shown in Figure 3b. The high value of *tan* may be impurities, crystal defects, etc. [50].

### 3.4. Electrical AC Conductivity of Li-Doped ZnO Nanoparticles

**Figure 4:**
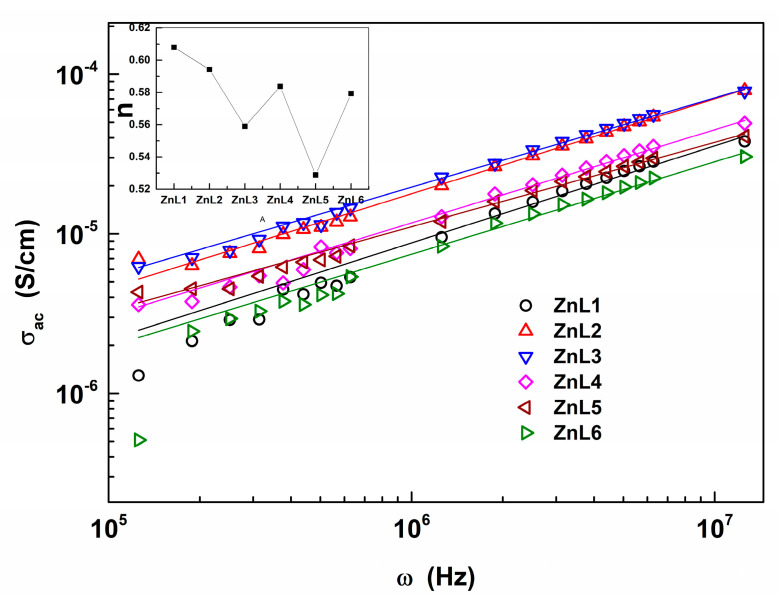
Double logarithmic plots of *σ_ac_* vs. *ω* for each sample. The linear fits (solid lines) through the data (symbols) validate the Equation (9). The inset shows the variation of *n*, the exponent in the Equation (9), concerning the studied samples.

Figure 4 illustrates AC conductivity (*σ_AC_*) against the frequency (*ω*) at different but fixed samples. The *σ_AC_* is calculated using a relation *σ_AC_ = ωε*_1_*ε*_0_*tanδ* [46] with *ε*_1_ and *ε*_0_ as the real part of the complex dielectric constant and permittivity of free space, respectively. As is evidenced by this figure, there is a linear relationship between the logarithmic *σ_ac_* and logarithmic *ω*, which indicates the power-law variation between *τηε σ_ac_* and *ω*. This relation has been proposed and widely used to fit the *σ_AC_*(*ω*). According to Ref. [51], the power law (Jonscher’s law) is given by the universal dynamic response at frequencies well below the lattice vibrational frequency as:

(9) $$\sigma_{AC}=\sigma_{DC}\left(1+{\left(\frac{\omega }{\omega_{p}}\right)}^{n}\right)$$

where *σ_DC_* is the DC conductivity, the characteristic frequency *ω_p_* corresponds to the activation energy of the DC conductivity or the onset of the AC conductivity or a typical hopping frequency of the ions that contribute to the conductivity [52], and *n* is the fractional exponent that measures the degrees of correlation among moving ions [53]. The exponent *n* takes the values between 0 ≤ *n* ≤ 1 when a hopping of charge carriers is involved as a translational motion with a sudden hopping. By contrast, the *n* > 1 case pertains to localized hopping without leaving the neighborhood [54]. In the present study, although the *n* values decrease from 0.61 to 0.51, there are some fluctuations in this value concerning the samples. Despite these alterations in *n*, a common attribution can be made on the magnitude of *n* for its values less than unity in all samples. A hopping of charge carriers is involved as a translational motion with a sudden hopping [54].

### 3.5. Optical Studies of Li-Doped ZnO Nanoparticles

**Figure 5:**
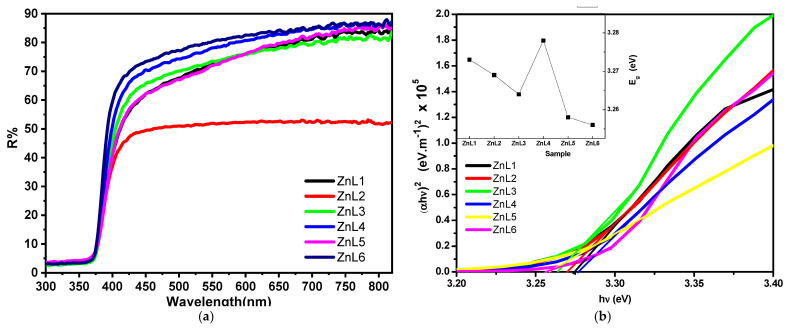
(**a**) Diffused reflection spectra of pure and Li-doped ZnO nanostructures. (**b**) Tauc’s plots to estimate the optical band gap values. Inset: This panel depicts the *E_g_* values for the pure and Li-doped ZnO samples.

UV-Vis spectroscopy measured all the samples’ optical diffused reflectance in the 300–1800 nm wavelength region that embraces both the UV and visible regions. As shown in Figure 5a, an abrupt increase in reflectance occurs between 370 nm–430 nm. This abrupt change is called the absorption edge, from which one can calculate the optical band gap (*E_g_*) as described in the proceeding discussion. This sudden change may be attributed to the absorption of the electrons of level 1 S_h_ (fundamental state) to 1 S_e_ (excitation state) of excitonic transition in ZnO nanoparticles [55]. Then its variation became gradual in the visible region. The reflectance in the visible region is 80% for all the samples except ZnL2, which exhibits 50% reflectance. The highest reflectance is observed in the case of ZnL6. The optical band gap (*E_g_*) can be calculated using Tauc’s relation [56]:

(10) $$\alpha h\nu =A{\left(h\nu -E_{g}\right)}^{z}$$

where *α* is the absorption coefficient, defined as absorbance divided by thickness, *hν* is incident photon energy, A is a constant, and *z* is an empirical constant whose values are ½ and 2 for direct and indirect optical bandgap transitions, respectively. Figure 5b represents how the variation of (*αhυ*)^2^ as a function of *hυ* is obtained using Equation (10). The direct bandgap value (*E_g_*) is obtained using a linear fit to the high slope data by extrapolating it onto the abscissa as marked by straight lines. Thus, the *E_g_* values are calculated and plotted against each sample (Figure 5b inset). As seen in the inset, the *E_g_* drops compared to pure-ZnO values, e.g., changes vary from 3.273 eV (for pure ZnO, i.e., ZnL1) to 3.256 eV (for pure ZnO, i.e., ZnL1) (for ZnL6). The prepared ZnL4 exhibits a higher *E_g_* than any present samples, 3.278 eV. Regardless of this variation, it is understood that the bandgap’s narrowing down with doping is a well-known phenomenon in semiconductors. The reduction of *E_g_* may be due to the introduction of energy levels near the valence band by the dopant ions [57].

### 3.6. Raman Study of Li-Doped ZnO Nanoparticles

**Figure 6:**
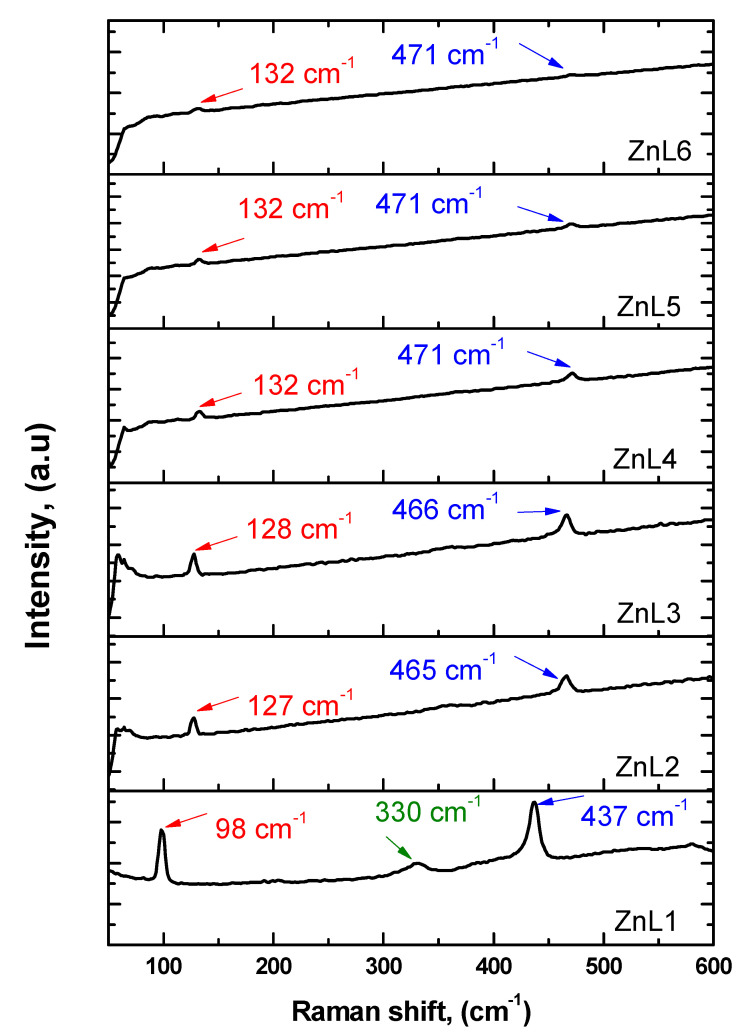
Raman spectrum of pure and Li-doped ZnO nanostructures.

Figure 6 illustrates the room temperature Raman spectra of all the samples. It highlights that two peaks at ~98 cm^−1^ and 437 cm^−1^ for the ZnL1 sample correspond to acoustic combinations in the low wavenumber region. In ZnL2 and ZnL3 samples, these two peaks shifted to ~127 cm^−1^ and 465 cm^−1^. In other samples, i.e., ZnL4, ZnL5, and ZnL6, these two peaks shifted to 132 cm^−1^ and 471 cm^−1^, regardless of the sample details. The two peaks at ~98 cm^−1^ and 437 cm^−1^ which are commonly observed are the characteristic modes of the wurtzite ZnO and are indexed to be *E*_2_*^low^* and *E*_2_*^high^*, respectively [54]. As is known from the literature [58,59,60], neighboring ions move opposite to each other in the plane perpendicular to the c-axis in the E_2_ phonon modes. Therefore, the total displacement and net polarization are zero, and *E*_2_*^low^* and *E*_2_*^high^* modes are non-polar phonon modes, which are the characteristic peaks of the hexagonal wurtzite phase. Furthermore, *E*_2_*^low^* and *E*_2_*^high^* modes mainly involve the motion of oxygen atoms and vibrations of the Zn sub-lattice, respectively. The peak at 330 cm^−1^, only observed in the ZnL1 sample, is ascribed to the ZnO multiphonon process and assigned to the *E*_2_*^high^−E*_2_*^low^* mode [61]. This peak has not been observed in any other present samples, which means these samples have no role in multiphonon processes and no second-order Raman modes [62]. Another exciting feature is the suppression of the peaks corresponding to *E*_2_*^low^* and *E*_2_*^high^* as the Li-content increases (i.e., from sample ZnL1 to ZnL6). This could be due to the poor crystallinity of the samples [60]. By contrast, a shift in the position of *E*_2_*^high^* mode towards high wave numbers as the Li-content increases is due mainly to the distortion of the lattice and defects induced by the doping of Li-ions [63].

### 3.7. Electrocatalysis Study of Li-Doped ZnO Nanoparticles

**Figure 7:**
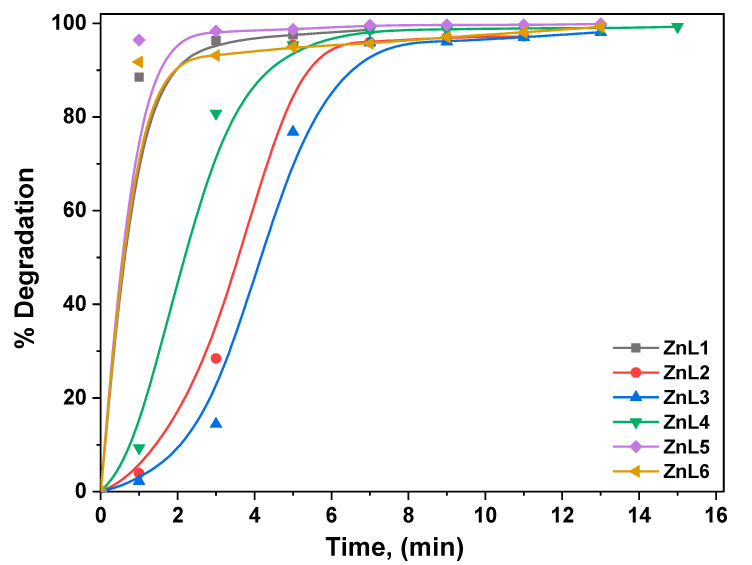
The % of E.C. degradation of RhB in the presence of ZNCM nanostructures.

**Figure 8:**
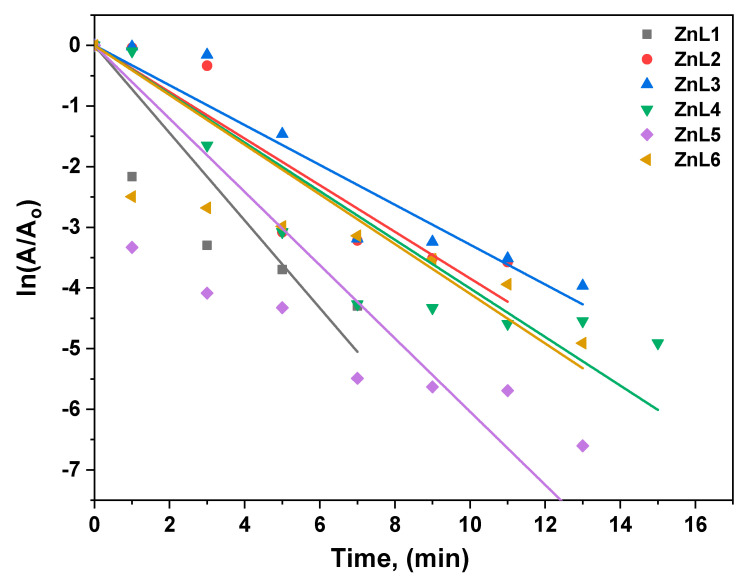
Kinetic study of E.C. degradation of RhB in the presence of pure and Li-doped ZnO nanostructures.

RhB dye was investigated as a model organic species for the ZnL. (1–6) electrode, and its degradation via E.C. was investigated. A voltage of 10 V was supplied between the photoanode and cathode in the single-cell reactor to assist charge carrier transfer via the external circuit. The data gathered and recorded throughout the degradation process show a decrease in organic species absorbance with reaction time, indicating a reduction in organic species concentration. In the presence of six different ZnL. samples, E.C. destroyed RhB, as illustrated in Figure 7. The results show that the ZnL5 showed 100% in 3 min electrocatalytic degradation, attributed to the lower bandgap and crystallite size. As previously observed, E.C. degradation of RhB followed pseudo-first-order kinetics (Figure 8). For RhB degradation, the following pseudo-first-order kinetics were calculated [64,65,66]:

*ln(A/A_o_)* = −*kt* (11)

The efficiency of degradation was calculated using the formula shown below [61]:

% *of degradation* = *(A_o_* − *A_t_/A_o_)* × 100% (12)

**Figure 9:**
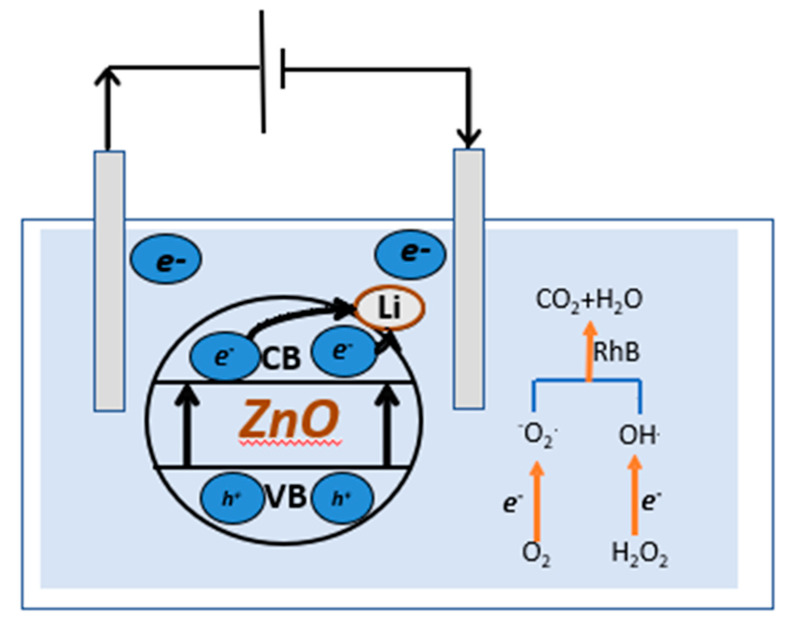
E.C. degradation mechanism.

At the start, there is an absorbance of *A_o_*. The absorbance, at different times, is denoted by *A_t_*; the value of the rate constant, *K*, and the duration of the reaction, *t*, are all given. It has been shown that a gradient at the ZnL. surface effectively separates the resulting charge carriers [64]. After traveling an electric path longer than the *E_g_* on the ZnL., electrons from the valence band (V.B.) are stimulated into the conduction band, resulting in V.B. holes. During the degradation process, electrons move via the external circuit to counter electro, helping produce extremely reactive superoxide anion radicals (O_2_). Water oxidation by valence band holes yields hydroxyl (O.H.) radicals, which react with organic contaminants to mitigate their negative effects. In the case of ZnL. nanoparticles, the primary degradation species are O_2,_ which produced holes (depending on the bonding of the catalyst with the pollutant). Electrocatalytic oxidation mechanisms have a cumulative effect on ZnL. degradation. RhB [67,68] photodegradation utilizing ZnL. in the presence of E.C. is predicted to occur via the following pathways, as illustrated in the equations and Figure 9. During the photoelectrocatalysis, with anodic polarization, the excited electrons from the valence band to the ZnO conduction band are directed to the external circuit to the counter electrode, improving the separation of charges.

Meanwhile, the photogenerated holes on the ZnO surface area can react with H_2_O and form OH^.^ radicals. In addition, the O_2_ adsorbed on the surface of the counter electrode can react with the injected electron, and the O_2_ adsorbed on the surface of the ZnO can respond with the photoinduced electrons. Thus, the recombination of charges is reduced, resulting in a greater production of active species, such as ^−^O_2_ and OH^.^, improving the photo electrocatalytic efficiency.

O_2_+ e^−^ → ^−^O_2_ (13)

^−^O_2_^.^+ 2H^+^ + 2e^−^ → H_2_O_2_ (14)

H_2_O_2_ + e^−^ → ^.^OH +^−^OH (15)

(^.^OH/ ^−^O_2_^.^) + RhB → products (16)

(h^+^/ H_2_O_2_) + RhB → CO_2_+ H_2_O (17)

## 4. Conclusions

In summary, we have prepared Li-doped ZnO nanostructures using the combustion method. According to X-ray diffraction measurements, these samples crystallize into a typical hexagonal wurtzite ZnO structure with a *P63mc* space group. The SEM micrographs conceal all samples’ nanosphere bundles and their aggregates. The measured capacitance concerning frequency has estimated the variation of dielectric constant, dielectric loss, and AC conductivity against AC electric field frequency. The dielectric constant value at low frequencies is high due to an enhanced space-charge polarization, which validates Koop’s phenomenological theory. The variation of AC conductivity follows Jonscher’s law in all the samples. Such a validation gives the magnitude of an exponent *n*, which reveals that the conduction is involved as a hopping of charge carriers involved as a translational motion with a sudden hopping. Analysis of the optical diffused reflectance spectra unveiled the direct bandgap values in the range 3.273 eV (for pure ZnO, i.e., ZnL1) to 3.256 eV (for high Li-doped ZnO). Narrowing down the bandgap with doping, a well-known semiconductor phenomenon, is attributed to introducing energy levels near the valance band due to the addition of Li-ions to ZnO. Raman spectra have been recorded and analyzed for the observed Raman modes. These modes are the characteristic peaks of the hexagonal wurtzite phase, and there is a slight shift of the peaks corresponding to these modes as the Li-content increases in the ZnO. Electrocatalytic studies have investigated the prepared samples’ performance in wastewater purification.

## Figures and Tables

**Table 1:** The sample codes of ZnO nanostructures with different doping concentrations of Lithium Li+ ions.

Samples	Sample Codes
Zinc acetate + 30 mL distilled water	ZnL1
Zinc acetate + glycine + 30 mL distilled water	ZnL2
Zinc acetate + glycine + 30 mL distilled water + 0.001 LiNO_3_	ZnL3
Zinc acetate + glycine + 30 mL distilled water + 0.01 LiNO_3_	ZnL4
Zinc acetate + glycine + 30 mL distilled water + 0.1 LiNO_3_	ZnL5
Zinc acetate + glycine + 30 mL distilled water + 0.5 LiNO_3_	ZnL6

**Table 2:** Structural parameters of prepared samples such as average crystallite size (D), micro-strain (*ε*), dislocation density (*δ*), lattice parameters *a* and *c*, and volume of the unit cell. Numbers in the parenthesis are error values in the respective parameter.

Samples	D (nm)	ε (10^−3^)	δ (10^15^ lines.m^−2^)	a (Å)	c (Å)	V (Å^3^)
ZnL1	22.81(23)	5.07(5)	1.99(4)	3.229	5.177	46.746
ZnL2	24.10(20)	4.81(4)	1.79(3)	3.233	5.181	46.898
ZnL3	23.49(23)	4.94(5)	1.92(4)	3.238	5.189	47.116
ZnL4	23.31(19)	4.96(4)	1.94(3)	3.223	5.168	46.491
ZnL5	20.70(24)	5.61(6)	2.42(6)	3.240	5.193	47.211
ZnL6	26.31(23)	4.40(4)	1.53(3)	3.227	5.173	46.652

## Data Availability

Data will be made available on request.
